# Supplementary material for: A thermodynamic cycle to predict the competitive inhibition outcomes of an evolving enzyme
Source: bioRxiv. 2025 Feb 7:2025.02.03.636225. Preprint. [Version 1] doi: 10.1101/2025.02.03.636225 (PMC11838402; doi:10.1101/2025.02.03.636225)

A thermodynamic cycle to predict the competitive inhibition outcomes of an evolving enzyme

Ebru Cetin,† Haleh Abdizadeh,‡ Ali Rana Atilgan, Canan Atilgan*

Faculty of Engineering and Natural Sciences, Sabanci University

***Correspondence:** Canan Atilgan, Faculty of Natural Sciences and Engineering, Sabanci University, Tuzla 34956 Istanbul, Turkey, E-mail: canan@sabanciuniv.edu

† Present address: Department of Chemistry & Biochemistry, University of Arizona
‡ Present address: Department of Strategic Development, University of Twente

Figure S1 | Probability densities for the energy distributions in DHF bound, **(a)** F153S and **(b)** S153F runs.

(a)

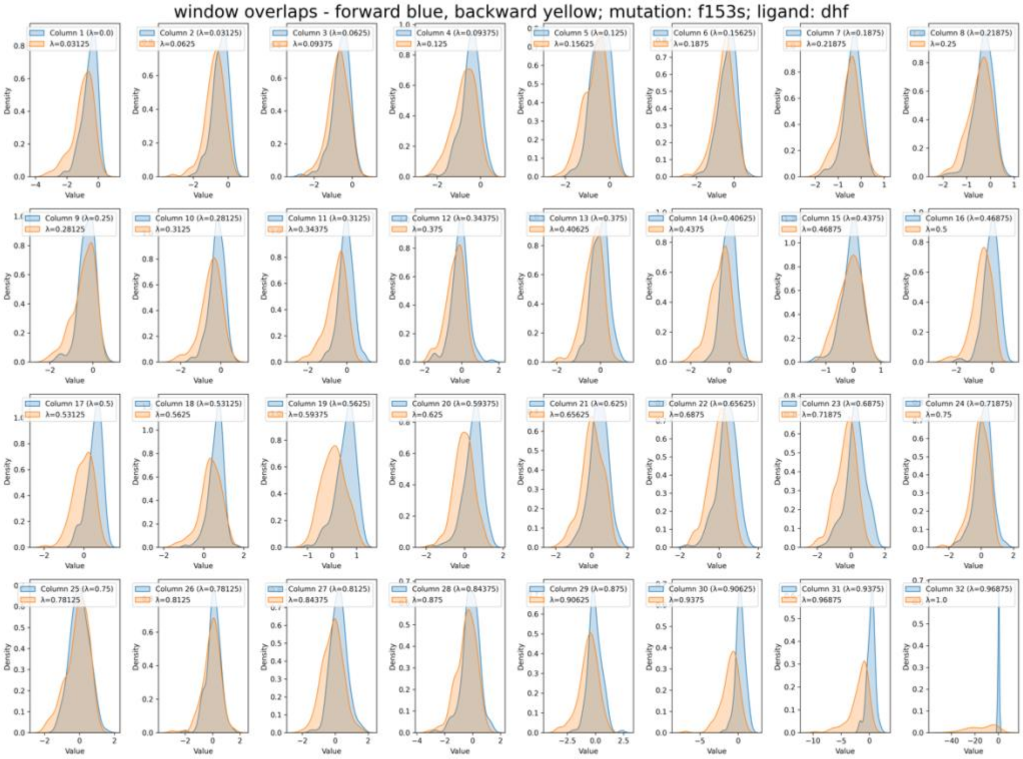

(b)

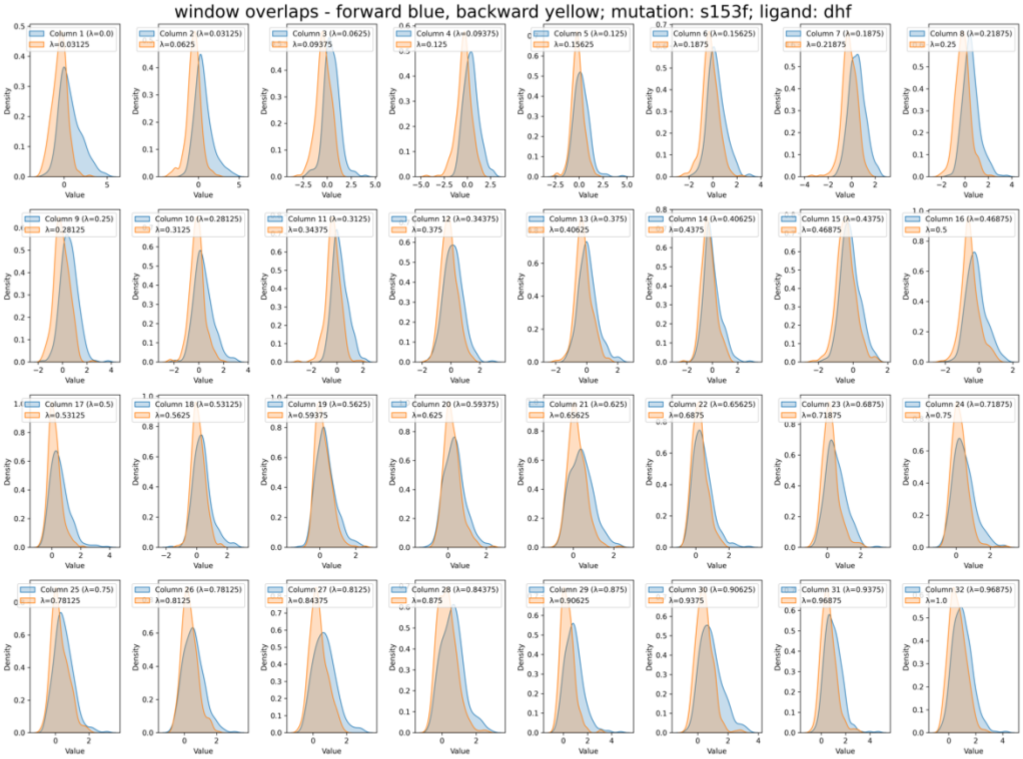

Supplement: Supplement 1 [file media-1.pdf]
